# Effects of plasma magnesium and prolactin on quantitative ultrasound measurements of heel bone among schizophrenic patients

**DOI:** 10.1186/1471-2474-11-35

**Published:** 2010-02-17

**Authors:** Jenn-Huei Renn, Nan-Ping Yang, Pesus Chou

**Affiliations:** 1 Community Medicine Research Center, National Yang-Ming University, Taipei, Taiwan, ROC; 2 Institute of Public Health, National Yang-Ming University, Taipei, Taiwan; 3 Department of Orthopaedic Surgery, Yuli Veterans Hospital, Veterans' Affairs Commission, Executive Yuan, Yuli Town, Taiwan; 4 Department of Orthopaedic Surgery, Tao-Yuan General Hospital, DOH, Executive Yuan, Tao-Yuan, Taiwan

## Abstract

**Background:**

Osteoporosis is a bone disease that can reduce both bone mass and bone strength. It can cause serious fractures of bones, along with causing significant and even devastating physical, psychological and financial consequences for patients and their family members. Many reports have revealed that the prevalence of decreased bone density is higher in schizophrenic patients than in the non-psychological diseased population. The previous report of our group revealed that chronic schizophrenia patients have poorer BUA levels since they were young as compared to the general community population. Hyperprolactinemia and antipsychotics are reported to be among the risk factors for osteoporosis in chronic schizophrenic patients.

**Methods:**

93 schizophrenic patients with severely poor adjusted BUA values and 93 age and gender matched patients with normal adjusted BUA values from a previous survey study were selected. Data were collected via questionnaires and via reviews of antipsychotic medications. Blood samples were drawn, and serum levels of prolactin, estradiol, testosterone, magnesium, calcium, phosphate, osteocalcin, Cross-linked N-teleopeptide of type I collagen (NTX), thyroid hormone and parathyroid hormone were checked. The association between BUA levels and serum levels of the above items, along with the type of received antipsychotic medication, was evaluated.

**Results:**

There was no significant association found between reduced BUA levels and serum prolactin, calcium, phosphate, osteocalcin, NTX, thyroid stimulating hormone and parathyroid hormone levels. There was also no association between BUA levels and types of currently received antipsychotics. There was no association between BUA levels and menstruation condition in female patients. Hypermagnesemia had a borderline association with classical and combined (classical and atypical) antipsychotic medications in male patients. Nevertheless, hypermagnesemia is a significant protective factor of reduced BUA levels in female patients. Hyperprolactinemia had a significant association with classical and combined antipsychotic medications in female patients. Hyperprolactinemia, however, provides a protective effect on reduced BUA levels in male patients. There was no significant association found between serum prolactin level and the type of antipsychotic medication received.

**Conclusions:**

The results of this study are in contrast with literature that has reported an association between bone mass and serum prolactin levels, serum magnesium levels and type of received antipsychotics. Further study to investigate the pathophysiological process and the association between bone mass and serum prolactin level, serum magnesium level and specific antipsychotics is necessary.

## Background

Osteoporosis is a skeletal disorder that is characterized by compromised bone strength. It is typically thought to be age-related [1]. In an osteoporotic bone, bone strength and bone stiffness are so severely deteriorated that fractures can occur with minimal trauma. Either the decrease of bone density, bone quality or both can cause osteoporosis. The NIH Consensus Development Conference defined osteoporosis as, "a disease characterized by low bone strength, leading to enhanced bone fragility and a consequent increase in fracture risk" [2]. Fractures caused by osteoporosis can have significant and even devastating physical, psychological and financial consequences on patients and their families [3,4].

Schizophrenia is a chronic, severe and disabling brain disorder. It is a devastating disorder for most people who are afflicted and is very costly for families and society. Onset of symptoms typically occurs in young adulthood [5], between the ages of 15 and 25. The prevalence in the United States is about 1% [6]. Schizophrenia occurs in all societies regardless of class, color, religion and culture. There are, however, some variations in terms of incidence and outcomes for different groups of people. The prevalence is around 0.5 to 1% from country to country [5,7-16]. Schizophrenia patients are significantly more likely to have one or more chronic medical conditions. Hypothyroidism, chronic obstructive pulmonary disease, diabetes, hepatitis C, fluid/electrolyte disorders and nicotine abuse/dependence have been reported to have higher incidence rates in schizophrenia patients [17-20]. Osteoporosis is another disease that has been reported to have higher prevalence in schizophrenia patients than in the general population [21].

Early onset of poor BUA levels in chronic schizophrenia patients without a further BUA level decrease during the aging process was reported in an earlier survey conducted by our group [22]. A decrease of BUA levels that is found during menopausal transition in the female general population is not found in female schizophrenic patients. This study is designed to evaluate the association between BUA levels and different types of received antipsychotics (classical or atypical), serum prolactin, calcium, phosphate, osteocalcin, Cross-linked N-teleopeptide of type I collagen (NTX), thyroid stimulating hormone (TSH), parathyroid hormone level and menstruation condition.

## Methods

### Study population

93 chronic schizophrenic patients with severely poor adjusted BUA levels, as determined by heel QUS-II measurement, from our previous bone mass survey study [22] agreed to enter the study. Another group of 93 age and gender matched patients with normal adjusted BUA levels, as determined by heel QUS-II measurement, in the same study were selected. Reduced bone mass is defined as a T score equal or less than -2.5 computed from BUA data of QUS II. These 186 chronic schizophrenic patients were all aged 20 years and older. They were all inpatients of the Department of Psychiatry at Yuli Veterans Hospital in Taiwan, ROC, from 2003 to 2004. As of 2007, all were still inpatients of the unit. Psychiatrists at Yuli Veterans Hospital diagnosed all patients according to DSM-IV criteria. Exclusion criteria included any other medical diseases or use of medications known to develop osteoporosis or to affect bone mass. Patients having received their current antipsychotics less than one year or having received medication for osteoporosis were excluded as well. The study was approved by the medical ethics policy of the Institutional Review Board Committee at Yuli Veterans Hospital (serial No. 94-09-04A). As per this policy, all subjects were informed of the use of their medical information and of the blood drawing, and gave their consent.

### Laboratory tests, questionnaire and history review

All patients received a questionnaire regarding their general data, major disease history, current medications and previous medication history. Menstruation cycle condition was also included in the questionnaire for female patients. Psychiatrists of Yuli Veterans Hospital reviewed the medical history of these patients in regards to the antipsychotic medications. Medical history in the inpatient and outpatient departments of Yuli Veterans Hospital were both included. These patients were classified into classical, mixed group (neuroleptic medication combines classical and atypical antipsychotics) and atypical group according to the current antipsychotics they were receiving. Blood samples were drawn in the morning. Samples were processed and analyzed as soon as possible in the clinical laboratory of Yuli Veterans Hospital. The laboratory has passed ISO9001^® ^and ISO15189^® ^qualifications. Serum prolactin and TSH levels were measured with chemiluminescent immunoassay (Beckman Access^®^). Serum calcium, magnesium and phosphate levels were measured using Dade Behring RxL Max^®^. Other tests were performed at Union Clinical Laboratory in Taiwan. Blood samples were frozen at -78°C during preservation and transportation. Serum Osteocalcin level was measured using the electrochemiluminescence method of Roche using Elecsys 2010^®^. Serum PTH, estradiol and testosterone levels were measured using the chemiluminescence method of Bayer using ADVIA Centaur^®^. Serum NTX was measured with the ELISA method of Osteomark^® ^using BIO-RAD CODA^®^.

### Statistical analysis

All subjects were classified into two groups according to BUA levels: normal BUA levels or severely reduced BUA levels. Four logistic regression models were used step by step. Each model distinguished between genders. The first logistic regression model analyzed the association between BUA levels and serum levels of prolactin, calcium, phosphate, osteocalcin, NTX, TSH and parathyroid hormone, along with types of currently received antipsychotics by gender. The association between the type of antipsychotic medication currently received and serum levels of prolactin, calcium, phosphate, osteocalcin, NTX, TSH and parathyroid hormone were analyzed by the second logistic regression model. Interactions of serum prolactin levels and serum estradiol/testosterone levels were also tested in this model. The association between serum prolactin levels and serum levels of calcium, phosphate, osteocalcin, NTX, TSH and parathyroid hormone by gender was analyzed in the third logistic regression model. The fourth regression model analyzed the association between BUA levels and menstruation, serum levels of prolactin, estradiol and testosterone. Interaction between menstruation and serum prolactin was analyzed in this model as well. All statistical calculations were conducted using the Stata 10/SE system for Mac.

## Results

The demographic data is shown in Additional file 1. There were 48 normal BUA level and 48 reduced BUA level male patients. In addition, there were 45 normal BUA level and 45 reduced BUA level female patients. Similar mean age and an equal number of cases in both normal BUA level and reduced BUA level of both genders confirmed the age and gender matched parity. The mean BUA value of the male normal and reduced BUA level groups was 91.21 (±1.56) and 49.25 (±0.92), respectively. The mean BUA value of the female normal and reduced BUA level groups was 98.53 (±2.5) and 51.27 (±0.82), respectively.

The results, shown in Additional file 1, reveal that the male reduced BUA level patients had lower serum prolactin (26.91 ± 3.29 ng/ml vs. 39.70 ± 4.69 ng/ml), testosterone (436.48 ± 29.56 ng/dl vs. 476.13 ± 27.54 ng/dl), phosphate (3.30 ± 0.09 mg/dl vs. 3.44 ± 0.08 mg/dl), magnesium (2.13 ± 0.03 mg/dl vs. 2.19 ± 0.03 mg/dl), osteocalcin (16.26 ± 0.96 ng/ml vs. 17.00 ± 0.88 ng/ml), parathyroid hormone (21.23 ± 1.77 pg/ml vs. 28.68 ± 2.23 pg/ml) and thyroid stimulating hormone (1.63 ± 0.13 mIU/ml vs. 1.75 ± 0.13 mIU/ml) levels. The male patients with reduced BUA levels had higher serum estradiol (18.35 ± 1.95 pg/ml vs. 16.62 ± 1.49 pg/ml), calcium (8.82 ± 0.05 mg/dl vs. 8.78 ± 0.05 mg/dl) and NTX (10.14 ± 1.00 nM BCE vs. 9.90 ± 0.46 nM BCE) levels. The male patients with reduced BUA levels displayed more cases of having received atypical antipsychotics than the normal BUA level patients (29 cases vs. 25 cases).

The female reduced BUA level patients had lower serum estradiol (37.65 ± 7.88 pg/ml vs. 51.95 ± 8.94 pg/ml), testosterone (46.14 ± 2.41 ng/dl vs. 55.25 ± 3.62 ng/dl), calcium (8.73 ± 0.06 mg/dl vs. 8.78 ± 0.07 mg/dl), phosphate (3.71 ± 0.07 v mg/dl s. 3.85 ± 0.10 mg/dl), magnesium (2.10 ± 0.03 mg/dl vs. 2.20 ± 0.03 mg/dl), NTX (11.22 ± 0.71 nM BCE vs. 11.38 ± 0.59 nM BCE) and parathyroid hormone (16.09 ± 1.19 pg/ml vs. 29.88 ± 1.96 pg/ml) levels. The female reduced BUA level patients had higher serum prolactin (62.83 ± 8.62 ng/ml vs. 55.60 ± 8.01 ng/ml), osteocalcin (18.6 ± 1.52 ng/ml vs. 16.09 ± 1.19 ng/ml) and thyroid stimulating hormone (1.96 ± 0.18 mIU/ml vs. 1.56 ± 0.14 mIU/ml) levels. The female reduced BUA level patients and normal BUA level patients had the same pattern of antipsychotic usage.

Additional file 2 shows the results of the logistic regression model with BUA level as the dependent variable. Independent variables include serum levels of prolactin, estradiol, testosterone, calcium, phosphate, magnesium, THS, parathyroid hormone, osteocalcin and NTX, along with the current antipsychotic medication. The only significant association found was between BUA levels and serum prolactin levels in male patients. Hyperprolactinemia is a protective factor (odds ratio = 0.30, 95% confidence interval: 0.11 ~ 0.81) of reduced BUA levels. In female patients, the only significant association was between serum magnesium levels and BUA levels. Hypermagnesemia was found to be a protective factor of reduced BUA levels (odds ratio = 0.25, 95% confidence interval: 0.09 ~ 0.66). Atypical antipsychotic use results in an odds ratio larger than one in male patients but smaller than one in female patients.

Additional file 3 shows the results of the logistic regression model with antipsychotics as the dependent variable and serum levels of prolactin, estradiol, testosterone, calcium, phosphate, magnesium, thyroid stimulating hormone, parathyroid hormone, osteocalcin and NTX as independent variables. Only the serum prolactin level was found to be significantly associated with the received antipsychotics in female patients, but it was not found to be significant in male patients. The association between antipsychotic medications and serum magnesium levels was found to be borderline significant in male patients (p = 0.074), but was not significant in female patients. There was no significant association between the type of antipsychotic medication and serum levels of estradiol, testosterone, calcium, phosphate, TSH, parathyroid hormone, osteocalcin and NTX in either gender. Interaction between serum prolactin levels and sex hormones (estradiol in females and testosterone in males) was tested in the same model and was not found to be significant.

Additional file 4 reveals the results of the association between serum prolactin levels and serum levels of estradiol, testosterone, calcium, phosphate, magnesium, TSH, parathyroid hormone, osteocalcin and NTX.

There was no male patient with a serum estradiol level lower than the normal range in the sample; therefore, estradiol was dropped in all of the logistic regression models. There was only one male patient with serum phosphate levels and serum NTX levels higher than normal range in the study; therefore, serum levels of phosphate and NTX were dropped in the logistic regression model. There were only two male patients with serum TSH levels higher than normal; therefore, the serum TSH level was dropped in the logistic regression model. In female patients, there was only one case with serum testosterone levels lower than the normal range, so the serum testosterone level was dropped in the logistic regression models.

Another logistic regression model was generated with BUA level as the dependent variable. Age, serum magnesium level, serum prolactin level and antipsychotic medication were the independent variables. Menstruation status was also an independent variable in the female group. The results are shown in Additional file 5. The only significant factor found was high serum magnesium level, which was found to be a protective factor of reduced BUA level in female patients. In male patients, the only significant factor found was high serum prolactin level, which was also found to be a protective factor of reduced BUA levels. Interaction between gender and serum prolactin level was insignificant, as was the interaction between gender and serum magnesium level. There was also no significant interaction between serum prolactin level and menstruation status.

The results reveal that the serum magnesium level is significantly associated with BUA level condition in female patients and is insignificant in male patients. Serum prolactin level is significantly associated with BUA level condition only in male patients but is not significant in female patients. The effect of serum magnesium level and serum prolactin level on BUA level is not the same in different genders. There is a borderline association between serum magnesium level and present antipsychotic medication in male patients, and there is a significant association between serum magnesium level and BUA levels only in female patients. In contrast, the serum prolactin level is significantly associated with the current antipsychotic medication in female patients and significantly associated with BUA level in male patients.

## Discussion

In this study, hypermagnesemia displays a borderline association with classical or mixed antipsychotics in male patients. Hypermagnesemia, however, provides a significant protective effect on BUA levels in female patients. Constipation is a common medical problem in chronic schizophrenia patients admitted in the chronic care unit of Yuli Veterans Hospital. Therefore, many patients receive magnesium oxide as a laxative. This may be the cause of the high serum magnesium levels. Magnesium has been reported to possibly inhibit calcification of osteoids in *in vitro *experiments. Hypermagnesemia inhibits mineralization of collagen *in vivo *and *in vitro *[23] and was reported to cause abnormalities in metaphyses in neonates [24]. According to these reports, elevated serum magnesium levels should correlate with reduced bone mass. Nevertheless, hypermagnesemia is a significant protection factor on reduced BUA levels in this study. This result is in contrast with previously reported results. The mechanism of hypermagnesemia being a protective factor of reduced BUA levels in female patients should be investigated further. Possible confounders may exist that were not considered in this study.

Hyperprolactinemia is significantly associated with classic or mixed antipsychotics in female patients in this study. Hyperprolactinemia provides a significant protective effect on reduced BUA levels in male patients. This, however, is also in contrast with previous reports in literature.

Many research projects have reported that hyperprolactinemia correlates with poor bone mass in schizophrenia patients. This is caused either by hyperprolactinemia itself or by a process of resulting hypogonadism. Hyperprolactinemia has been reported with classic antipsychotic drugs [25]. Hyperprolactinemia caused by the administration of many kinds of neuroleptics was suspected to suppress gonadotropins and gonadal hormones [26-28]. Chlorpromazine, the oldest antipsychotic medication, has been reported to disturb calcification of bones [29]. Higher doses of prolactin-raising antipsychotic medications are associated with higher serum prolactin levels. Higher doses of prolactin-raising medications are associated with hyperprolactinemia and lower BMD [28]. Serum prolactin level is negatively correlated with BMD value in the lumbar spine and hip, but it is only significant over the hip area. Neither testosterone nor estradiol was shown to influence the relationship between serum prolactin level and BMD to a significant level in a 16-subject study [30]. Low BMD values and hypogonadism were reported in young schizophrenic women with hyperprolactinemia who were treated with prolactin-raising antipsychotics [31].

Hui *et al. *reported mean BMD values more than one standard deviation lower in hyperprolactinemic women [1]. Coss *et al. *reported that prolactin might have a direct inhibitory effect on osteoblasts [32].

On the contrary, there are also many projects reporting different results. Halbreich *et al. *reported serum prolactin levels do not correlate with BMD in women but negatively correlate with BMD in men [26]. Becker *et al. *performed a study involving 26 female, premenopausal schizophrenic subjects. In their work, BMD values were similar in the hyperprolactinemia and normal prolactin level subjects both at the lumbar and femoral neck region. Nevertheless, the adjusted bone speed of sound was significantly lower in the hyperprolactinemia group at the radius and phalanx [33]. No statistical correlation between circulating prolactin and bone speed of sound has also been reported [34,35]. Greenspan *et al. *reported that BMD correlated with the duration of hyperprolactinemia but not with the degree of hyperprolactinemia [36]. The Z-score of BMD value is correlated with the duration of treatment in high prolactin level patients but not in normal prolactin level patients [37]. Elevated serum prolactin levels are not associated with BMD in vertebral or hip regions in a prospective study. Hyperprolactinemia, however, has a direct impact on the rate of bone metabolism, both in bone formation and in bone resorption [38]. Lean *et al. *reported that there is no evidence that antipsychotic-induced hyperprolactinemia is an independent risk factor for osteoporosis in schizophrenia patients [39].

Many research projects have reported that hyperprolactinemia induced hypogonadism is correlated with poor bone mass in schizophrenia patients. Hyperprolactinemia and the subsequent hypogonadal state are a couple of the reported causes of low bone mass in schizophrenia patients [25,31,37,40-42]. Some projects, however, have reported that prolactin has a direct effect on bone and is not related to hypogonadal state and amenorrhea [26,27,34,35,43]. There is no association between hyperprolactinemia with serum estradiol levels and serum testosterone levels in this study. There is also no association between antipsychotic medication and serum estradiol levels in female and serum testosterone levels in male patients.

Atypical antipsychotics were reported to contribute to less osteoporosis and have fewer hyperprolactinemia side effects than classic neuroleptics [44]. Some atypical antipsychotics, like Risperidone, however, have been reported to be associated with hyperprolactinemia and osteoporosis [33,45-47]. Hyperprolactinemia has not been previously reported as being a protective factor of reduced BUA levels in male patients, as it is in this study. Further study is necessary to clarify the association between exactly prescribed antipsychotics and bone density and to find the pathophysiological process between hyperprolactinemia and severe bone mass loss.

There is no significant association between the type of prescribed antipsychotics and bone mass condition in this study. The type of antipsychotic medication prescribed is associated with hyperprolactinemia in female patients. Nevertheless, hyperprolactinemia does not associate with bone mass condition in females and is a protective factor for reduced bone mass in male patients. Hypermagnesemia is a protective factor for reduced BUA level in female patients but is borderline associated with antipsychotic medication in male patients. The association between hypermagnesemia, hyperprolactinemia, antipsychotic medication and reduced BUA level is modified by gender. That may be the reason there is no significant association between the type of antipsychotic drugs and BUA level condition. The pathophysiological processes of the effects of magnesium and prolactin on bone need further study to explore this issue.

## Conclusions

In this study, hyperprolactinemia and hypermagnesemia do not associate with types of antipsychotics. Hyperprolactinemia was found to be a protective factor of reduced BUA levels in male patients, and hypermagnesemia was found to be a protective factor in female patients. Bone metabolism markers do not correlate with serum prolactin levels and type of antipsychotic. There is no correlation between BUA level condition and serum sex hormone levels. Interaction between serum sex hormone levels and prolactin levels also does not exist. There is no significant association between serum calcium and phosphate levels and BUA levels in schizophrenia patients. There is also no significant association between serum calcium, phosphate level and type of antipsychotic medication.

Some of the results are not as the same as in previous reports. Confounders, such as exact medication, not included in this study may be the reason for the dissimilar results. Modulating factors other than the reported risk factors may also exist. Ethnic difference is another possible reason for the different results.

Use of ultrasonographic assessments instead of DXA scans and the assessment of bone mass at only one location are two of the limitations of this study. The role of ultrasound in diagnosing osteoporosis and accessing risk of fracture needs further study. Nevertheless, it is still a portable and inexpensive method of bone mass screening [48]. Case control studies have their limitations in studying pathophysiological processes. Dosage of antipsychotics, duration of treatment and physical activity were not included, and these are other limitations. The pathophysiological process is still important in schizophrenic patients since the prevalence of non-traumatic fractures in chronic schizophrenic patients has been reported to be about 25% [21]. Prospective comprehensive studies of bone dynamics in individuals with first-episode schizophrenia, as well as in patients treated with various medications, are needed in order to characterize the problem(s) and to develop relevant treatment and prevention strategies [49].

## Competing interests

The authors declare that they have no competing interests.

## Authors' contributions

JHR conducted and performed the whole project, NPY carried out the data analysis and developed the report tables, and PC designed and supervised the whole project.

## Pre-publication history

The pre-publication history for this paper can be accessed here:

http://www.biomedcentral.com/1471-2474/11/35/prepub

## Supplementary Material

Additional file 1 **Table S1**. Demographic data with the descriptive statistical result of serum prolactin, estradiol, testosterone, calcium, phosphate, magnesium, osteocalcin, NTX, PTH, TSH level, and current antipsychotic medication Click here for file

Additional file 2 **Table S2**. Comparison of BUA level status by serum prolactin, estradiol, testosterone, calcium, phosphate, magnesium, TSH, PTH, osteocalcin, NTX level and present antipsychotic medication in both genders by univariate analysis Click here for file

Additional file 3 **Table S3**. Comparison of present antipsychotic medication by serum prolactin, estradiol, testosterone, calcium, phosphate, magnesium, TSH, PTH, osteocalcin and NTX level in both genders by univariate analysis Click here for file

Additional file 4 **Table S4**. Comparison of serum prolactin level by serum estradiol, testosterone, calcium, phosphate, magnesium, TSH, PTH, osteocalcin, NTX level and present antipsychotic medication in both genders by univariate analysis Click here for file

Additional file 5 **Table S5**. Logistic regression model of severely poor QUS value in both genders Click here for file

## References

[B1] Hui SL Slemenda CW Johnston CC Jr Age and bone mass as predictors of fracture in a prospective study J Clin Invest 1988 81 1804 1809 10.1172/JCI113523 3384952 PMC442628

[B2] NIH Consensus Statement. Osteoporosis prevention, diagnosis, and therapy 2000 17 1 1 36 11525451

[B3] Consensus conference: Osteoporosis JAMA 1984 252 799 802 10.1001/jama.252.6.799 6748181

[B4] Cummings SR Rubin SM Black D The future of hip fractures in the United States. Numbers, costs, and potential effects of postmenopausal estrogen Clin Orthop Relat Res 1990 163 166 2302881

[B5] Castle D Wessely S Der G Murray RM The incidence of operationally defined schizophrenia in Camberwell, 1965-84 Br J Psychiatry 1991 159 790 794 10.1192/bjp.159.6.790 1790446

[B6] Regier DA Narrow WE Rae DS Manderscheid RW Locke BZ Goodwin FK The de facto US mental and addictive disorders service system. Epidemiologic catchment area prospective 1-year prevalence rates of disorders and services Arch Gen Psychiatry 1993 50 85 94 8427558 10.1001/archpsyc.1993.01820140007001

[B7] Kirkbride JB Boydell J Ploubidis GB Morgan C Dazzan P McKenzie K Murray RM Jones PB Testing the association between the incidence of schizophrenia and social capital in an urban area Psychol Med 2008 38 1083 1094 10.1017/S0033291707002085 17988420

[B8] Fearon P Kirkbride JB Morgan C Dazzan P Morgan K Lloyd T Hutchinson G Tarrant J Fung WL Holloway J Incidence of schizophrenia and other psychoses in ethnic minority groups: results from the MRC AESOP Study Psychol Med 2006 36 1541 1550 10.1017/S0033291706008774 16938150

[B9] Smith GN Boydell J Murray RM Flynn S McKay K Sherwood M Honer WG The incidence of schizophrenia in European immigrants to Canada Schizophr Res 2006 87 205 211 10.1016/j.schres.2006.06.024 16905294

[B10] Kirkbride JB Fearon P Morgan C Dazzan P Morgan K Tarrant J Lloyd T Holloway J Hutchinson G Leff JP Heterogeneity in incidence rates of schizophrenia and other psychotic syndromes: findings from the 3-center AeSOP study Arch Gen Psychiatry 2006 63 250 258 10.1001/archpsyc.63.3.250 16520429

[B11] Boydell J van Os J McKenzie K Murray RM The association of inequality with the incidence of schizophrenia--an ecological study Soc Psychiatry Psychiatr Epidemiol 2004 39 597 599 10.1007/s00127-004-0789-6 15300368

[B12] Boydell J Van Os J Lambri M Castle D Allardyce J McCreadie RG Murray RM Incidence of schizophrenia in south-east London between 1965 and 1997 Br J Psychiatry 2003 182 45 49 10.1192/bjp.182.1.45 12509317

[B13] Boydell J van Os J McKenzie K Allardyce J Goel R McCreadie RG Murray RM Incidence of schizophrenia in ethnic minorities in London: ecological study into interactions with environment BMJ 2001 323 1336 1338 10.1136/bmj.323.7325.1336 11739218 PMC60671

[B14] Allardyce J Boydell J Van Os J Morrison G Castle D Murray RM McCreadie RG Comparison of the incidence of schizophrenia in rural Dumfries and Galloway and urban Camberwell Br J Psychiatry 2001 179 335 339 10.1192/bjp.179.4.335 11581114

[B15] Takei N Lewis G Sham PC Murray RM Age-period-cohort analysis of the incidence of schizophrenia in Scotland Psychol Med 1996 26 963 973 10.1017/S0033291700035297 8878329

[B16] Gupta S Murray RM The relationship of environmental temperature to the incidence and outcome of schizophrenia Br J Psychiatry 1992 160 788 792 10.1192/bjp.160.6.788 1617362

[B17] Carney CP Jones L Woolson RF Medical comorbidity in women and men with schizophrenia: a population-based controlled study J Gen Intern Med 2006 21 1133 1137 10.1111/j.1525-1497.2006.00563.x 17026726 PMC1831667

[B18] Goldman LS Medical illness in patients with schizophrenia J Clin Psychiatry 1999 60 Suppl 21 10 15 10548136

[B19] Leucht S Burkard T Henderson J Maj M Sartorius N Physical illness and schizophrenia: a review of the literature Acta Psychiatr Scand 2007 116 317 333 10.1111/j.1600-0447.2007.01095.x 17919153

[B20] Green AI Canuso CM Brenner MJ Wojcik JD Detection and management of comorbidity in patients with schizophrenia Psychiatr Clin North Am 2003 26 115 139 10.1016/S0193-953X(02)00014-X 12683263

[B21] Abraham G Friedman RH Verghese C de Leon J Osteoporosis and schizophrenia: can we limit known risk factors? Biol Psychiatry 1995 38 131 132 10.1016/0006-3223(95)00062-L 7578648

[B22] Renn JH Yang NP Chueh CM Lin CY Lan TH Chou P Bone mass in schizophrenia and normal populations across different decades of life BMC Musculoskeletal Disorders 2009 10 1 10.1186/1471-2474-10-1 19118498 PMC2642755

[B23] Shipley PG Kramer B Howland J Studies upon Calcification in vitro Biochem J 1926 20 379 387 16743669 10.1042/bj0200379 PMC1251724

[B24] Cumming WA Thomas VJ Hypermagnesemia: a cause of abnormal metaphyses in the neonate AJR Am J Roentgenol 1989 152 1071 1072 2705341 10.2214/ajr.152.5.1071

[B25] Hummer M Huber J Hyperprolactinaemia and antipsychotic therapy in schizophrenia Curr Med Res Opin 2004 20 189 197 10.1185/030079903125002865 15006013

[B26] Halbreich U Palter S Accelerated osteoporosis in psychiatric patients: possible pathophysiological processes Schizophr Bull 1996 22 447 454 8873295 10.1093/schbul/22.3.447

[B27] Levinson DF Simpson FM Antipsychotic drug side effects 1987 Washington, DC: American Psychiatric Association

[B28] Meaney AM Smith S Howes OD O'Brien M Murray RM O'Keane V Effects of long-term prolactin-raising antipsychotic medication on bone mineral density in patients with schizophrenia Br J Psychiatry 2004 184 503 508 10.1192/bjp.184.6.503 15172944

[B29] Kutsuma K Bone histomorphometric study of the ilium in psychiatric patients with longterm administration of anti-psychiatric drugs Nippon Seikeigeka Gakkai Zasshi 1993 67 583 590 8409629

[B30] Abraham G Halbreich U Friedman RH Josiassen RC Bone mineral density and prolactin associations in patients with chronic schizophrenia Schizophr Res 2003 59 17 18 10.1016/S0920-9964(01)00321-8 12413637

[B31] O'Keane V Meaney AM Antipsychotic drugs: a new risk factor for osteoporosis in young women with schizophrenia? J Clin Psychopharmacol 2005 25 26 31 10.1097/01.jcp.0000150223.31007.e0 15643097

[B32] Coss D Yang L Kuo CB Xu X Luben RA Walker AM Effects of prolactin on osteoblast alkaline phosphatase and bone formation in the developing rat Am J Physiol Endocrinol Metab 2000 279 E1216 1225 11093907 10.1152/ajpendo.2000.279.6.E1216

[B33] Becker D Liver O Mester R Rapoport M Weizman A Weiss M Risperidone, but not olanzapine, decreases bone mineral density in female premenopausal schizophrenia patients J Clin Psychiatry 2003 64 761 766 12934975 10.4088/jcp.v64n0704

[B34] Halbreich U Rojansky N Palter S Hreshchyshyn M Kreeger J Bakhai Y Rosan R Decreased bone mineral density in medicated psychiatric patients Psychosom Med 1995 57 485 491 8552740 10.1097/00006842-199509000-00011

[B35] Schlechte JA Sherman B Martin R Bone density in amenorrheic women with and without hyperprolactinemia J Clin Endocrinol Metab 1983 56 1120 1123 10.1210/jcem-56-6-1120 6404918

[B36] Greenspan SL Neer RM Ridgway EC Klibanski A Osteoporosis in men with hyperprolactinemic hypogonadism Ann Intern Med 1986 104 777 782 3706929 10.7326/0003-4819-104-6-777

[B37] Kishimoto T Watanabe K Shimada N Makita K Yagi G Kashima H Antipsychotic-induced hyperprolactinemia inhibits the hypothalamo-pituitary-gonadal axis and reduces bone mineral density in male patients with schizophrenia J Clin Psychiatry 2008 69 385 391 10.4088/JCP.v69n0307 18278991

[B38] Abraham G Paing WW Kaminski J Joseph A Kohegyi E Josiassen RC Effects of elevated serum prolactin on bone mineral density and bone metabolism in female patients with schizophrenia: a prospective study Am J Psychiatry 2003 160 1618 1620 10.1176/appi.ajp.160.9.1618 12944336

[B39] Lean M De Smedt G Schizophrenia and osteoporosis Int Clin Psychopharmacol 2004 19 31 35 10.1097/00004850-200401000-00006 15101568

[B40] Naidoo U Goff DC Klibanski A Hyperprolactinemia and bone mineral density: the potential impact of antipsychotic agents Psychoneuroendocrinology 2003 28 Suppl 2 97 108 10.1016/S0306-4530(02)00129-4 12650684

[B41] Klibanski A Neer RM Beitins IZ Ridgway EC Zervas NT McArthur JW Decreased bone density in hyperprolactinemic women N Engl J Med 1980 303 1511 1514 7432421 10.1056/NEJM198012253032605

[B42] Kartaginer J Ataya K Mercado A Abbasi A Osteoporosis associated with neuroleptic treatment. A case report J Reprod Med 1990 35 198 202 1968102

[B43] Ataya K Mercado A Kartaginer J Abbasi A Moghissi KS Bone density and reproductive hormones in patients with neuroleptic-induced hyperprolactinemia Fertil Steril 1988 50 876 881 2904890 10.1016/s0015-0282(16)60365-5

[B44] Bilici M Cakirbay H Guler M Tosun M Ulgen M Tan U Classical and atypical neuroleptics, and bone mineral density, in patients with schizophrenia Int J Neurosci 2002 112 817 828 10.1080/00207450290025833 12424822

[B45] Miller KK Management of hyperprolactinemia in patients receiving antipsychotics CNS Spectr 2004 9 28 32 15303078 10.1017/s1092852900002340

[B46] Hamner M The effects of atypical antipsychotics on serum prolactin levels Ann Clin Psychiatry 2002 14 163 173 12585566 10.1023/a:1021138603935

[B47] Howes OD Smith S Aitchison KJ Comment on Hyperprolactinaemia and antipsychotic therapy in schizophrenia Curr Med Res Opin 2004 20 1649 10.1185/030079904X4941 15462698

[B48] Krieg MA Barkmann R Gonnelli S Stewart A Bauer DC Del Rio Barquero L Kaufman JJ Lorenc R Miller PD Olszynski WP Quantitative ultrasound in the management of osteoporosis: the 2007 ISCD Official Positions J Clin Densitom 2008 11 163 187 10.1016/j.jocd.2007.12.011 18442758

[B49] Halbreich U Osteoporosis, schizophrenia and antipsychotics: the need for a comprehensive multifactorial evaluation CNS Drugs 2007 21 641 657 10.2165/00023210-200721080-00003 17630817

